# Progenitor-exhausted T cell as prognostic indicator in esophageal squamous cell carcinoma: illuminating their key contribution to tumor immunity

**DOI:** 10.3389/fimmu.2025.1659077

**Published:** 2025-09-26

**Authors:** Yi Liu, Hongwei Jiang, Zhang Fang, Bin Xu, Junjun Chen, Xiao Zheng, Renhao Geng, Lujun Chen

**Affiliations:** ^1^ Department of Tumor Biological Treatment, The Third Affiliated Hospital of Soochow University, Changzhou, Jiangsu, China; ^2^ Jiangsu Engineering Research Center for Tumor Immunotherapy, The Third Affiliated Hospital of Soochow University, Changzhou, Jiangsu, China; ^3^ Institute of Cell Therapy, The Third Affiliated Hospital of Soochow University, Changzhou, Jiangsu, China

**Keywords:** progenitor-exhausted CD8^+^ T cells, esophageal squamous cell carcinoma, multi-omics, immunotherapy, prognosis

## Abstract

**Background:**

Despite notable advances with immune checkpoint inhibitors (ICIs) in esophageal squamous cell carcinoma (ESCC), their clinical efficacy remains limited, largely due to CD8⁺T cell exhaustion. Among these, progenitor exhausted T cells (T_pex_) represent a key subset with stem cell–like features that sustain durable anti-tumor immunity.

**Methods:**

We applied multi-color immunohistochemistry (mIHC) to determine the spatial distribution and clinical significance of T_pex_ cells within the tumor microenvironment (TME) of ESCC. Publicly available single-cell RNA sequencing (scRNA-seq) datasets were further analyzed to characterize T_pex_ cell phenotypes, differentiation trajectories, and intercellular communication networks.

**Results:**

T_pex_ cells constituted a distinct subset of infiltrating CD8⁺T cells and represented a transitional stage of the exhaustion continuum. A higher degree of T_pex_ infiltration was significantly associated with improved overall survival in ESCC patients. Moreover, scRNA-seq data from patients treated with PD-1 blockade revealed that responders harbored markedly enriched T_pex_ populations compared with non-responders.

**Conclusion:**

Our findings identify T_pex_ cells as a critical prognostic and immunologically active CD8⁺T cell subset in ESCC. Their abundance and functional engagement are closely associated with favorable clinical outcomes and response to PD-1 blockade. Furthermore, their stem cell-like properties may be pivotal in shaping durable anti-tumor immunity and could provide novel therapeutic targets to enhance the efficacy of PD-1-based immunotherapy.

## Introduction

Esophageal cancer (EC) is one of the most commonly diagnosed gastrointestinal (GI) malignancies globally and ranks as the seventh leading cause of tumor-related mortality worldwide (1–3). ESCC accounts for over 90% of all EC cases, characterized by a dismal prognosis and high mortality rate, primarily due to the challenges in early detection and the scarcity of clinically validated therapeutic strategies (4–6). Endoscopic techniques, including mucosal and submucosal dissection, are considered standard-of-care approaches for patients presenting with early-stage disease (7). In patients with resectable and locally advanced tumors, the conventional approach comprises neoadjuvant chemoradiotherapy followed by surgical intervention (8). Nevertheless, close to 40% of patients experience disease recurrence despite existing interventions, highlighting a pressing demand for more potent and clinically effective therapeutic strategies (5, 9, 10).

The treatment landscape for locally advanced and metastatic esophageal malignancies has undergone significant transformation, primarily driven by advances in immunotherapy. Particularly, inhibitors targeting immune checkpoint molecules such as PD-1 and CTLA-4 have revolutionized clinical management approaches (11–13). As first-line treatment options for ESCC, both nivolumab and pembrolizumab have received regulatory approval, with clinical applications including monotherapy or combination regimens with chemotherapy. Additionally, multiple novel PD-1 inhibitors are undergoing active investigation in various clinical trial settings (14–18). The clinical benefit of PD-1 blockade is primarily mediated through the restoration of CD8^+^T cell functionality and the alleviation of immunosuppressive mechanisms operating within the TME (19, 20). As key cytotoxic effector cells, CD8^+^T cells mediate anti-tumor responses through cytokine secretion and direct tumor cell lysis (21). However, the progressive dysfunction of these cells—termed T-cell exhaustion—constitutes a substantial barrier to effective immunotherapeutic intervention and is widely recognized as a mechanism of resistance (22, 23). Chronic antigenic stimulation, characteristic of prolonged infections and neoplastic progression, contributes to the emergence of an exhausted T-cell phenotype with compromised functionality and heightened inhibitory receptor expression (24, 25).

T-cell exhaustion represents a dynamic and evolving process, resulting in notable functional and phenotypic diversity (26, 27). Among the exhausted populations, terminally exhausted T cells originate from a subset known as T_pex_ cells, which are generally marked by TCF1^+^, PD-1^+^, and CD8^+^ phenotypes (28, 29). These progenitor cells exhibit transcriptional programs reminiscent of early memory T cells, expressing genes such as TCF1 and surface markers including CCR7, IL7R, and CD62L (30, 31). Compared to terminally exhausted cells, T_pex_ cells show elevated expression of *SLAMF6*, *CXCR5*, and *BTLA*, with a notable absence of *TIM-3* expression (24, 32, 33). Notably, T_pex_ cells exhibit memory-associated characteristics, including self-renewal capability and the propensity to give rise to effector lineages, which have been consistently correlated with enhanced therapeutic efficacy of immune checkpoint blockade (ICB) (31, 34–36). Nevertheless, the overall effectiveness of ICIs remains limited, highlighting the need for novel therapeutic strategies that can robustly activate and maintain T_pex_ cells during PD-1/PD-L1-targeted immunotherapy.

Despite being linked to improved prognosis in cancers like B-cell lymphoma, melanoma, and lung cancer, the clinical implications of T_pex_ cell infiltration in ESCC have yet to be fully elucidated (29, 37, 38). scRNA-seq, an advanced transcriptomic approach for resolving cellular heterogeneity at high resolution, has revealed diverse immune cell subsets that orchestrate anti-tumor responses across different cancer types. An integrated scRNA-seq and mIHC approach was utilized to systematically investigate the localization patterns and prognostic value of T_pex_ cells within the ESCC TME.

Spatial profiling by mIHC enabled visualization of T_pex_ distribution, while scRNA-seq data provided insights into their phenotypic features, functional identity, and differentiation trajectory. Collectively, these findings elucidate the central function of T_pex_ cells in shaping immunotherapy outcomes and emphasize their dual potential as prognostic indicators and actionable targets in ESCC.

## Materials and methods

### Processing of Gene Expression Omnibus–derived scRNA-seq dataset

scRNA-seq data for ESCC were retrieved from the GEO database (accession number: GSE145370), comprising tumor samples from three stage II and four stage III ESCC patients. We followed the data processing workflow as described by Li H et al. (39), and the identification of canonical markers was also guided by original publication (40). T-cell subclustering was conducted using a resolution parameter of 1.0, followed by the extraction of CD8^+^T cells for downstream computational interrogation.

### Pseudotime analysis and trajectory inference

To explore the lineage trajectories of CD8^+^T-cell subsets, we conducted pseudotime analyses using both Monocle (version 2) and Slingshot, applying default parameters. The Seurat object was converted into a CellDataSet object for Monocle analysis. Dimensionality reduction was performed using the DDRTree algorithm, and cells were ordered along inferred developmental trajectories based on the HVGs identified earlier. For statistical modeling of gene expression variance, estimateSizeFactors, estimateDispersions, and dispersionTable functions were applied with default settings to characterize dynamic gene expression changes during differentiation.

### Patients and tumors

A human ESCC tissue microarray (TMA; Catalog no. HEsoS180Su05) was provided by Shanghai Outdo Biotech Co., Ltd. (Shanghai, China). The array included 105 primary tumor tissues and 75 adjacent normal tissues from patients aged 48 to 82 years. Survival status was determined through clinical follow-up. Following the exclusion of cases with incomplete survival information or tissue core loss, the final analysis incorporated 80 tumor specimens and 75 matched adjacent normal tissues. Survival data from these 80 patients were used for subsequent survival analysis. All procedures involving human specimens were reviewed and approved by the Clinical Research Ethics Committee of Outdo Biotech (Approval No. SHYJS-CP-1807012), ensuring compliance with institutional and national ethical requirements.

### mIHC and quantitative imaging analysis

mIHC was performed following the manufacturer’s protocol and as detailed in prior publications (41–43). Quantitative image acquisition and analysis were conducted using automated tools provided by PerkinElmer.

Whole-slide multispectral imaging and subsequent image analysis, including software and version details, were performed as previously described in our earlier study (43). Within this platform, fluorescent signals were spectrally unmixed, and each fluorophore was isolated into a separate channel and archived individually. DAPI staining was employed as a nuclear reference to guide accurate segmentation of cellular compartments, including nuclei, cytoplasm, and membranes.

Signal intensities for CK, KI67, CD8, TCF1, and PD-1 were co-registered with DAPI to generate binary expression masks for each biomarker. These masks were used to identify and quantify marker-positive cells. Tumor regions were delineated by pan-CK staining, which enabled precise discrimination between epithelial tumor cells and surrounding stromal compartments. Quantification of tumor cells was conducted based on the CK binary mask.

### Acquisition and preprocessing of HRA scRNA-seq data

scRNA-seq data from ESCC patients receiving anti-PD-1 therapy were obtained from the Genome Sequence Archive (GSA) at the National Genomics Data Center, China, under accession number HRA003312 from project PRJCA012636 (44). The dataset comprised tumor specimens (baseline and post-intervention) from seven ESCC patients undergoing neoadjuvant chemoimmunotherapy with PD-1 blockade. Among these patients, three exhibited complete responses, while four were classified as non-responders.

We followed the data processing workflow as described by the original authors, including all preprocessing steps and code implementation as provided in the corresponding publication and associated repositories. Cell clustering and subsequent analyses were conducted in Scanpy (version 1.8.1), with annotation informed by canonical markers and validated against reference profiles reported in the original study. Specifically, T cell–enriched populations were reclustered using the Leiden algorithm at a resolution of 1.5 to achieve finer granularity of cellular subtypes.

### Pathway enrichment analysis

To investigate the biological functions and signaling mechanisms associated with differential therapeutic responses, we applied R package clusterProfiler (version 4.8.3) to perform GO enrichment, KEGG pathway analyses, and GSEA. Annotations and hallmark gene sets were sourced from the MSigDB database (version 7.5.1). Pathways showing significant differences (*P* < 0.05) between complete responders (CR) and non-complete responders (NCR) were identified and visualized using bar plots and dot plots to illustrate enrichment trends.

### Cell–cell ligand–receptor communication analysis

Intercellular communication networks between T_pex_ cells and other immune cell populations were inferred using the CellChat R package (version 1.1.3) (41). The analysis was conducted on scRNA-seq datasets stratified by treatment response. The number and strength of inferred ligand–receptor interactions were quantified, and the global information flow of each signaling pathway was compared between CR and NCR groups. A minimum threshold of 10 cells per cell type was required for inclusion in the analysis. Significant signaling interactions, including those uniquely enriched in the CR group, were extracted for further investigation. Average expression levels of ligand–receptor pairs in CR-specific pathways were visualized using heatmaps generated by the ComplexHeatmap package (version 2.6.2) (42).

### Statistical analysis

To categorize patients into high- and low-infiltration cohorts according to T_pex_ cell abundance, the optimal cut-off threshold was identified using the survminer R package via the log-rank test. Kaplan–Meier survival analyses were performed in GraphPad Prism 9, and univariate and multivariate Cox regression analyses were conducted in R (version 4.2.2) within the RStudio environment. For validation purposes, survival analyses based on T_pex_-associated gene signatures in ESCC were executed using GEPIA2 (http://gepia2.cancer-pku.cn/#index), employing datasets from The Cancer Genome Atlas (TCGA).

Statistical methods were chosen according to the data distribution: normally distributed datasets were assessed with two-tailed, unpaired Student’s t-tests, whereas the Mann–Whitney U test was utilized for nonparametric comparisons between two groups. Statistical significance was denoted in figures as follows: *P < 0.05; **P < 0.01; ***P < 0.001; ns, not significant.

## Results

### T_pex_ cells reside in the TME of human ESCC tissues

**Figure 1:**
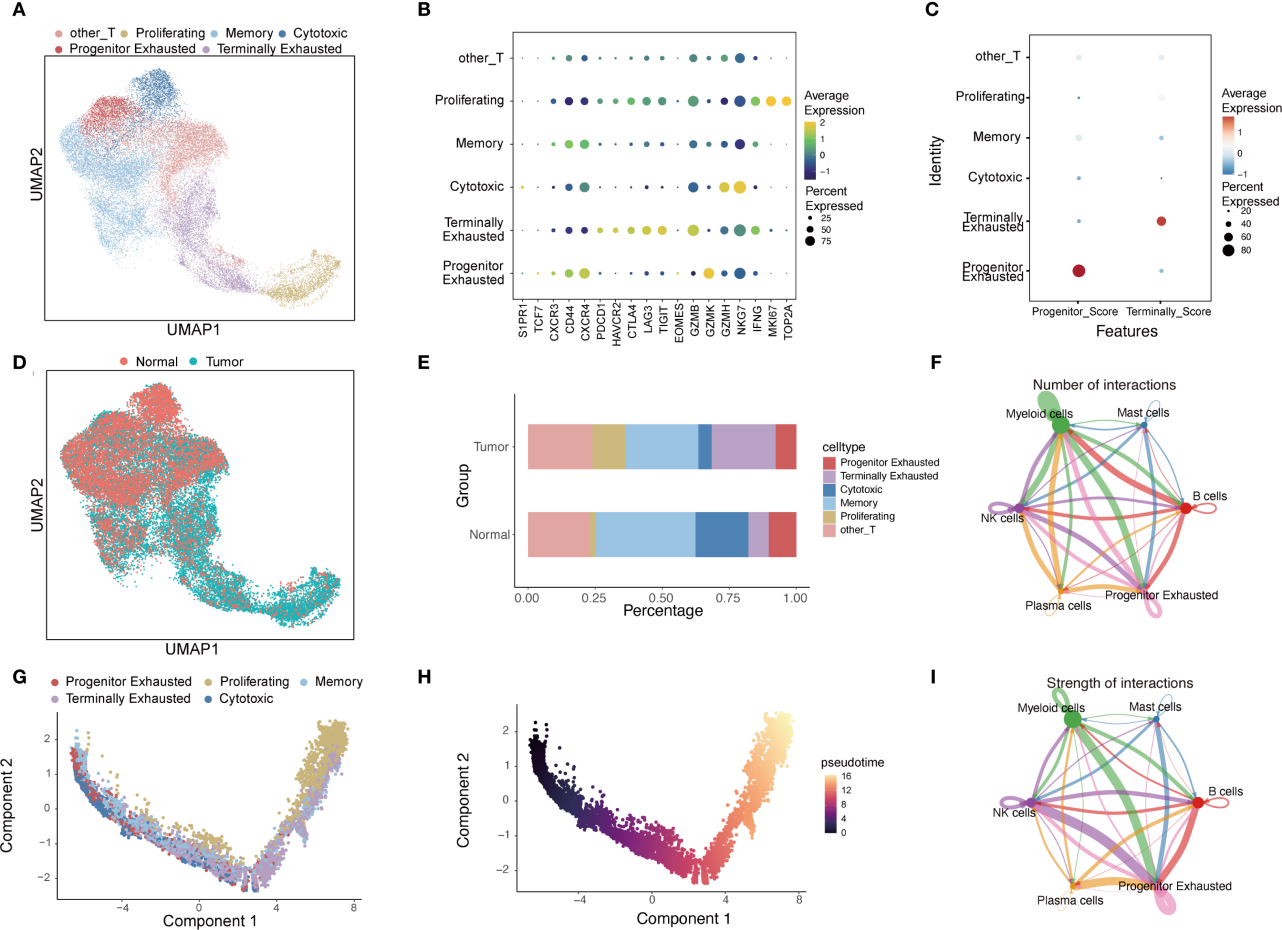
Pseudotime analysis of CD8^+^T cells infiltrating ESCC tissues. **(A)** UMAP plot depicting the clustering of CD8^+^T-cell subsets from dataset GSE145370. **(B)** Dot plot illustrating canonical marker genes delineating major CD8^+^T cell subsets. **(C)** Dot plot showing exhaustion signature scores across identified CD8^+^T cell subsets. Module scores were calculated using the *AddModuleScore* function based on markers defined by Sade-Feldman et al. **(D)** Tissue-specific distribution of CD8^+^T cells visualized on a UMAP plot, with normal (coral) and tumor (blue) tissue origins distinguished by color. **(E)** Proportions of CD8^+^T cell subtypes in adjacent normal versus tumor tissues. **(F, I)** Cell-cell interaction networks inferred by CellChat. **(G, H)** Pseudotime trajectories of CD8^+^T-cell subsets inferred by Monocle; each dot represents a single cell.

To elucidate the dynamic landscape and developmental hierarchy of CD8^+^T-cell subsets during ESCC progression, we analyzed a large-scale scRNA-seq dataset encompassing 102,611 cells. After reclustering, 29,073 CD8^+^T cells were identified and classified into six distinct subpopulations: T_pex_, terminally exhausted, cytotoxic, memory, and proliferative CD8^+^T cells, each defined by canonical gene expression signatures (**Figures 1A–C**). These CD8^+^T cells were derived from matched tumor and adjacent normal tissues of seven ESCC patients. Notably, T_pex_ cells were present in both tumor and normal adjacent tissues, with a marginally higher frequency detected in the normal tissue compartment (**Figures 1D, E**).

Pseudotime trajectory inference revealed that T_pex_ and memory T-cell clusters were positioned at early stages of the differentiation continuum, whereas proliferative and terminally exhausted subsets occupied later pseudotime states (**Figures 1G, H**), supporting the notion that T_pex_ represents an early exhausted progenitor population. Furthermore, intercellular communication analysis via CellChat indicated that T_pex_ cells exhibited robust interactions with myeloid populations within the ESCC TME (**Figures 1F, I**), underscoring their potential immunoregulatory role.

### Infiltration and prognostic significance of T_pex_ cells in ESCC tissues

**Figure 2:**
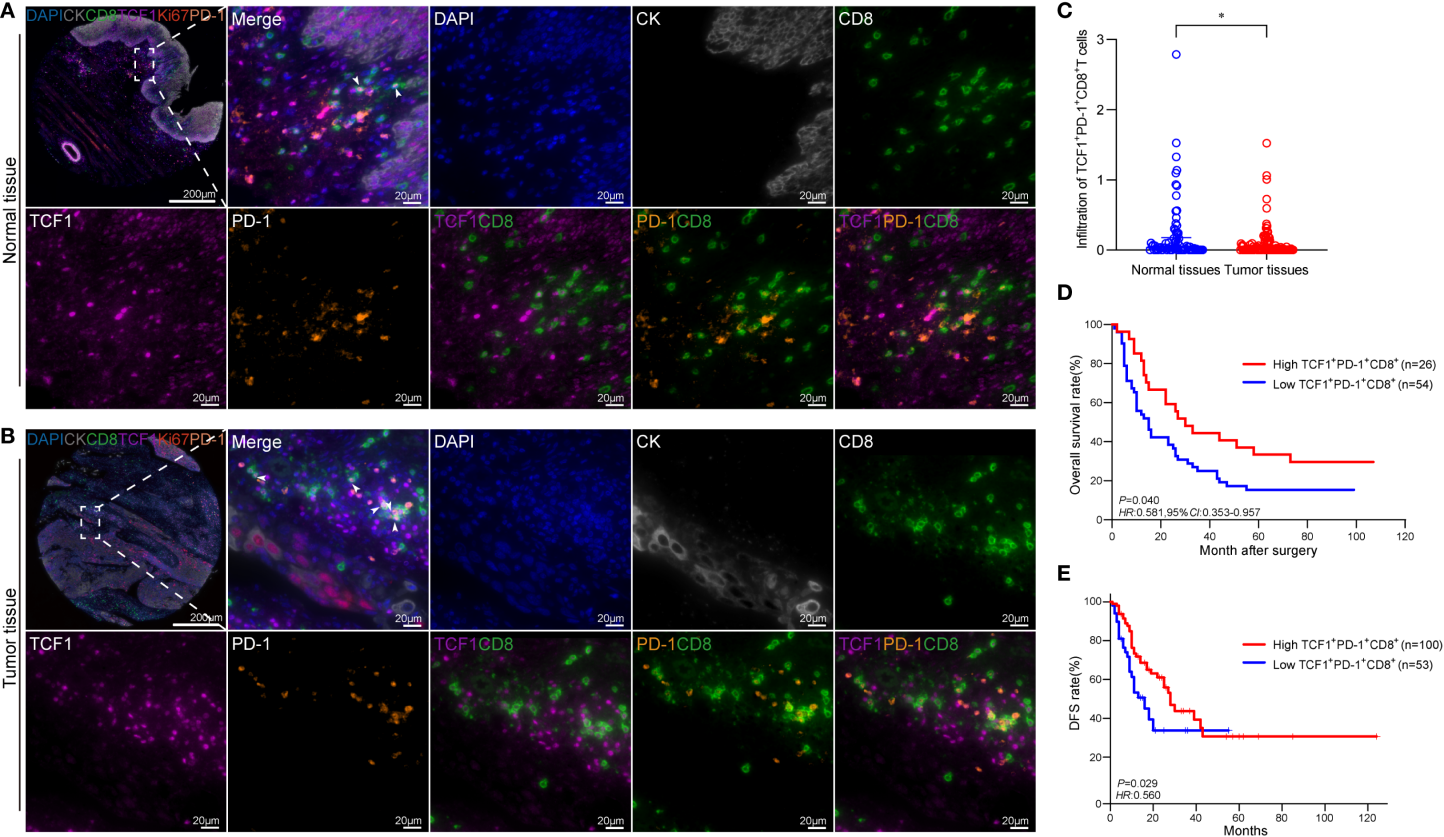
Distribution of T_pex_ infiltration in ESCC and adjacent normal tissues. **(A)** mIHC panoramic image and magnified region of adjacent normal tissue. Markers: CD8 (green), TCF1 (purple), PD-1 (orange), Ki67 (red), CK (gray). Scale bars: panoramic, 200 μm; magnified, 20 μm. Arrows indicate CD8^+^T_pex_-cell infiltration. **(B)** mIHC panoramic image and magnified region of tumor tissue. **(C)** Relative proportions of T_pex_ cells across tumor and matched normal tissues; Normal tissues exhibited markedly greater T_pex_ infiltration compared to tumor tissues (*P* < 0.05). **(D)** Kaplan–Meier curve showing OS stratified by low (blue) versus high (red) T_pex_ infiltration; significance assessed by log-rank test. **(E)** Kaplan–Meier survival curve of ESCC patients with high (red) versus low (blue) T_pex_ infiltration based on TCGA data. *P < 0.05.

mIHC was employed to simultaneously detect CK, CD8, TCF1, and PD-1 expression in ESCC tumor specimens and matched adjacent normal tissues (**Figures 2A, B**). Distinct CD8^+^T cell subpopulations were delineated based on the expression of TCF1 and PD-1, two key markers of differentiation states. TCF1^+^CD8^+^ cells are considered to possess stem-like properties, whereas PD-1^+^CD8^+^ cells are typically regarded as effector or exhausted populations. The TCF1^+^PD-1^+^CD8^+^ subset, co-expressing both markers, is identified as T_pex_ cells.

Quantitative analysis revealed a significant reduction in T_pex_-cell infiltration within tumor tissues relative to adjacent normal tissues (*P* < 0.05; **Figure 2C**). Survival analysis stratified by mIHC-defined T_pex_ abundance demonstrated that patients exhibiting high T_pex_ infiltration experienced significantly improved OS compared to those with low infiltration (cut-off = 0.05%, hazard ratio [HR] = 0.581, 95% confidence interval [CI]: 0.353–0.957, *P* < 0.05; **Figure 2D**). Validation in the TCGA-ESCC cohort demonstrated a statistically significant association between increased T_pex_ abundance and improved disease-free survival (DFS) (*P* < 0.05; **Figure 2E**), establishing its clinical relevance as a favorable prognostic indicator.

### Cox model analyses of T_pex_ infiltration and clinicopathological parameters in ESCC patients

Clinicopathological correlations were assessed in a cohort of 80 ESCC patients. Chi-square (*χ²*) analysis revealed a significant association between T_pex_-cell infiltration levels in tumor tissues and tumor size (*P* < 0.05), while no significant correlations were detected between T_pex_-cell infiltration and other clinicopathological parameters. A significant relationship was observed between PD-1^+^CD8^+^T cell infiltration and patient gender. However, infiltration levels of total CD8^+^T cells and TCF1^+^CD8^+^T cells did not demonstrate any substantial correlations with clinicopathological features (**Table 1**). Both univariate and multivariate Cox regression analyses were performed to evaluate prognostic factors for OS. As is shown, TNM stage remained an independent predictor in the multivariate model, with an HR of 3.410 (95% CI: 1.907–6.096, *P* < 0.001), confirming its strong association with poor prognosis. (**Table 2**).

**Table 1:** The correlation between ratio of infiltrating CD8^+^T cells, TCF1^+^CD8^+^T cells, PD-1^+^CD8^+^T cells, T_pex_ and clinical features of the patients.

Clinical parameters	Cases	Ratio of infiltrating CD8^+^T cells		*χ^2^*	*P* value	Ratio of infiltrating TCF1^+^CD8^+^T cells		*χ^2^*	*P* value	Ratio of infiltrating PD-1^+^CD8^+^T cells		*χ^2^*	*P* value	Ratio of infiltrating T_pex_ cells		*χ^2^*	*P* value	
		Low	High			Low	High			Low	High			Low	High			
Gender																		
Male	60	15	45	0.38	0.536^a^	37	23	0.84	0.359	40	20	4.44	0.035^*^	43	17	1.90	0.168	
Female	20	3	17			10	10			8	12			11	9			
Age(year)																		
≤60	23	3	20	1.66	0.198	13	10	0.07	0.797	14	9	0.01	0.920	15	8	0.08	0.782	
>60	57	15	42			34	23			34	23			39	18			
Tumor size(cm)																		
≤2.5	8	1	7	0.07	0.789^a^	2	6	2.77	0.096^a^	2	6	3.06	0.081^a^	2	6	5.33	0.021^a*^	
>2.5	72	17	55			45	27			46	26			52	20			
TNM stage																		
I+II	41	15	26	0.64	0.424	23	18	0.24	0.621	24	17	0.08	0.784	26	15	0.64		0.424
III+IV	39	11	28			24	15			24	15			28	11			
T stage																		
I+II	14	1	13	1.35	0.245^a^	5	9	3.72	0.053	7	7	0.71	0.400	8	6	0.36		0.551^a^
III+IV	66	17	49			42	24			41	25			46	20			
Pathological stage																		
I+II	58	14	44	0.07	0.787^a^	34	24	<0.01	0.970	37	21	1.26	0.261	38	19	0.06		0.802
III	22	4	18			13	9			11	11			16	7			

Bold signifies *P* < 0.05. “a”: Continuity Correction Chi-Square.

**Table 2:** Univariate analysis and multivariate analysis of factors affecting survival of ESCC patients.

Clinical parameters	Univariate		Multivariate	
	*HR*(95%*CI*)	*P* value	*HR*(95%*CI*)	*P* value
Sex(Male/Female)	1.77(0.94 ~ 3.32)	0.076	1.557(0.80 ~ 3.02)	0.191
Age(>60y/≤60y)	1.04(0.60 ~ 1.77)	0.900	1.621(0.89 ~ 2.96)	0.115
Tumor size(>2.5cm/≤2.5cm)	1.67(0.67 ~ 4.18)	0.270	1.433(0.53 ~ 3.87)	0.479
TNM stage(III+IV/ I+II)	3.27(1.92 ~ 5.58)	<0.001^*^	3.410(1.91 ~ 6.10)	<0.001^*^
Pathological stage(III/I+II)	0.96(0.56 ~ 1.64)	0.883	0.728(0.41 ~ 1.29)	0.279
Ratio of infiltrating of T_pex_ (High/Low)	0.62(0.36 ~ 1.06)	0.082	0.870(0.48 ~ 1.58)	0.647

### Prognostic value of T_pex_ infiltration in ESCC patients stratified by disease stage

**Figure 3:**
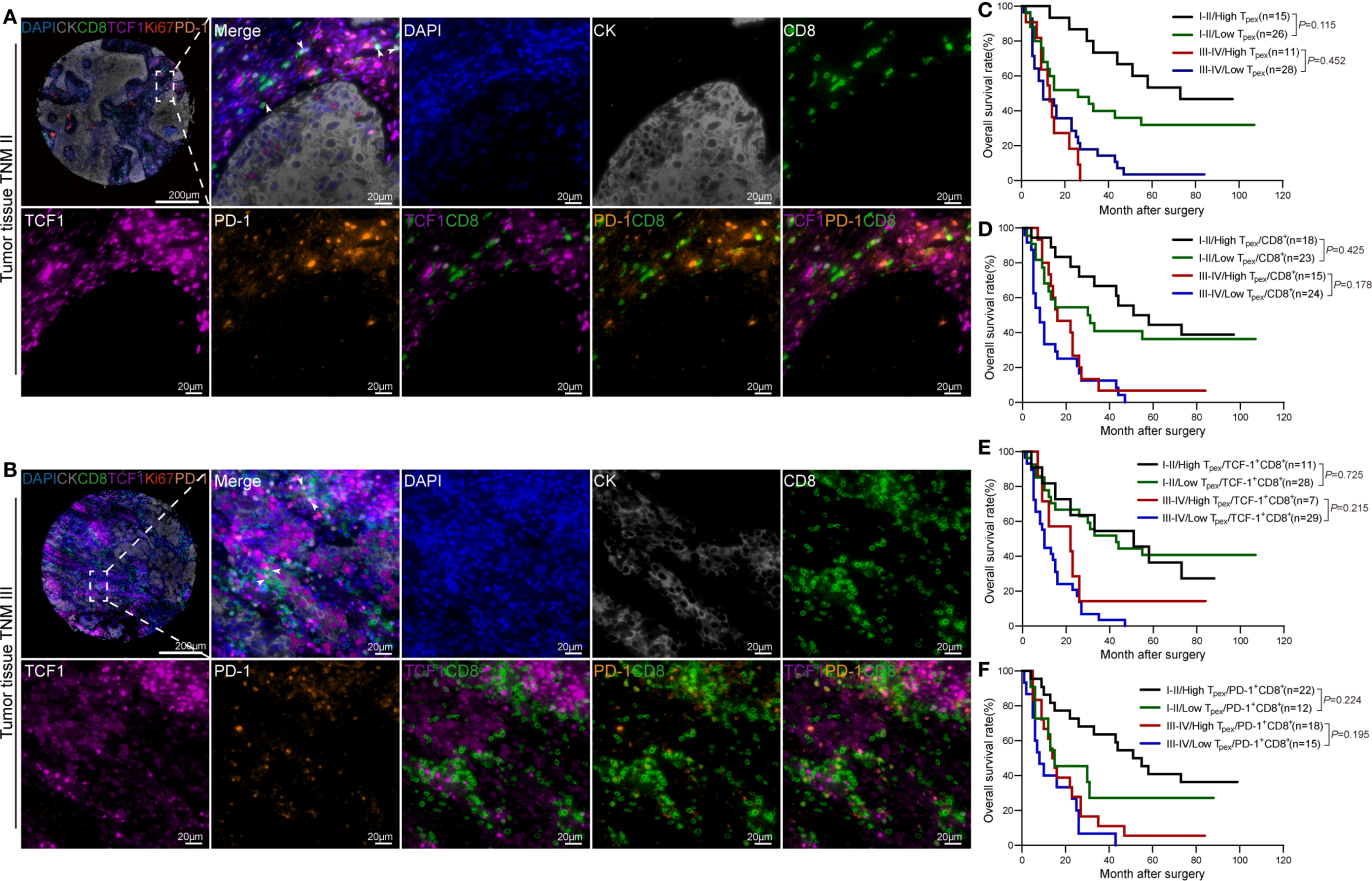
Prognostic analysis of T_pex_ infiltration in ESCC tissues at different stages. **(A)** Panoramic and magnified mIHC images of tumor tissue from a stage II ESCC patient. **(B)** Panoramic and magnified mIHC images of tumor tissue from a stage III ESCC patient. **(C)** Survival analysis based on T_pex_ infiltration levels in patients at TNM stages (I+II) and (III+IV). **(D)** Kaplan–Meier curves illustrating the prognostic impact of the T_pex_/CD8^+^T-cell ratio in TNM stage–stratified ESCC patients. **(E)** Kaplan–Meier curves illustrating the prognostic impact of the T_pex_/TCF1^+^CD8^+^T-cell ratio in TNM stage–stratified ESCC patients. **(F)** Kaplan–Meier curves illustrating the prognostic impact of the T_pex_ to PD-1^+^CD8^+^T-cell ratio in TNM stage–stratified ESCC patients.

Patients were stratified by TNM stage: those at stage I or II were assigned to the early-stage group, whereas stages III and IV were categorized as advanced-stage. Qualitative assessment of mIHC images showed differential T_pex_-cell infiltration between these groups, with representative examples presented (**Figures 3A, B**).

Early-stage patients (I+II) with abundant T_pex_-cell infiltration showed a promising trend for prolonged survival relative to those with minimal infiltration in our Kaplan–Meier survival analyses (**Figure 3C**). A similar pattern was observed when patients were stratified by the T_pex_ to total CD8^+^T-cell ratio, where a higher ratio correlated with marginally better OS (**Figure 3D**).

Within the advanced-stage cohort (stages III and IV), patients exhibiting higher ratios of T_pex_ to TCF1^+^CD8^+^T cells demonstrated a trend toward extended OS compared to those with lower ratios (**Figure 3E**). Furthermore, an increased T_pex_ to PD-1^+^CD8^+^T-cell ratio was similarly linked to modestly improved survival outcomes in this group (**Figure 3F**).

### Distinct cellular composition and transcriptional dynamics of T_pex_ cells in ESCC patients undergoing PD-1 therapy

Consistent with previous reports, we observed that T_pex_ infiltration correlated with improved clinical outcomes following ICB treatment in ESCC patients (44). We interrogated scRNA-seq data from project PRJCA012636. After rigorous quality control, 132,482 cells were annotated into 16 major populations using well-established marker gene profiles, encompassing neutrophils, monocytes, macrophages, B cells, endothelial and epithelial cells, plasmacytoid dendritic cells (pDCs), fibroblasts, pericytes, T cells, plasma cells, mast cells, and conventional dendritic cells (cDCs) (**Supplementary Figures 1A, B**).

To characterize immune heterogeneity relative to therapeutic response, we compared the proportions of major immune populations across pre- and post-treatment samples and between response groups (**Supplementary Figure 1C**). The cohort included three CR and four NCR. T cells constituted a predominant immune compartment, comprising over 50% of total cells in CR patients, a markedly higher proportion than in the NCR group.

Unsupervised clustering further subdivided the T-cell compartment into nine distinct subsets defined by their gene signatures, including CD8_T_rm_ (tissue resident memory), CD8_T_ex_ (terminally exhausted), CD8_MKi67 (proliferating), CD8_T_em_ (effector memory), CD8_T_pex_, CD4_T_ex_, CD4_T_cm_ (central memory), and regulatory T cells (T_reg_) (**Supplementary Figures 1D, E**). Among these, CD8_T_pex_ emerged as a key transitional population bridging functional and exhausted states.

**Figure 4:**
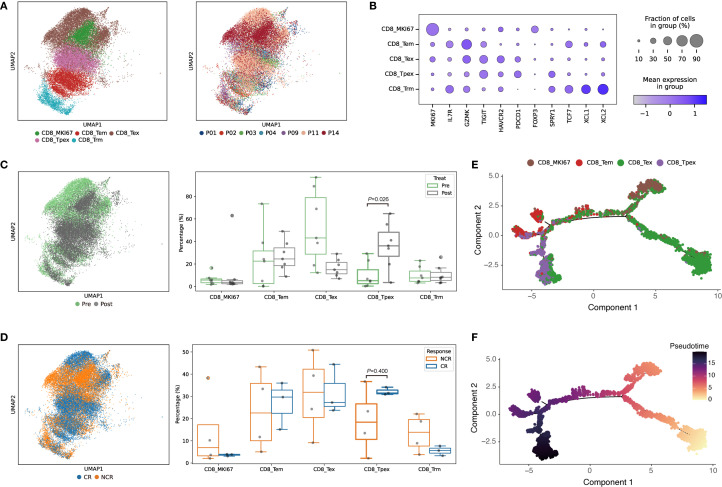
Single-cell transcriptomic profiling of CD8^+^T-cell states and differentiation dynamics in immunotherapy-treated ESCC patients. **(A)** Subclustering of CD8^+^T-cell subsets from dataset HRA003312. **(B)** Dot plot displaying percent and average expression of CD8^+^T-cell subclusters. **(C)** UMAP plot of CD8^+^T-cell subsets across pre-treatment (green) and post-treatment (gray) samples; boxplots show relative proportions per sample. **(D)** UMAP plot illustrating CD8^+^T-cell subset distribution across clinical response groups: CR (coral) and NCR (green); boxplots represent sample-wise proportions. **(E, F)** Pseudotime trajectory analysis of CD8^+^T-cell subsets by Monocle; cells are colored by subtypes.

We visualized CD8_T_pex_ cells within the CD8^+^T-cell compartment using UMAP embeddings, stratified by treatment time points and clinical response categories (**Figures 4A–D**). The relative abundance of T_pex_ cells significantly increased post-therapy and was consistently higher in the CR group compared to NCR.

Pseudotime trajectory analysis revealed two primary differentiation pathways originating from the CD8_T_pex_ cluster, diverging into either terminal exhaustion or proliferation branches (**Figures 4E, F**), consistent with our earlier findings (**Figure 1E**). This underscored the central role of T_pex_ cells in dictating divergent CD8^+^T-cell fates.

### Enhanced intercellular communication and pathway activation of T_pex_ cells in responders to PD-1 therapy

**Figure 5:**
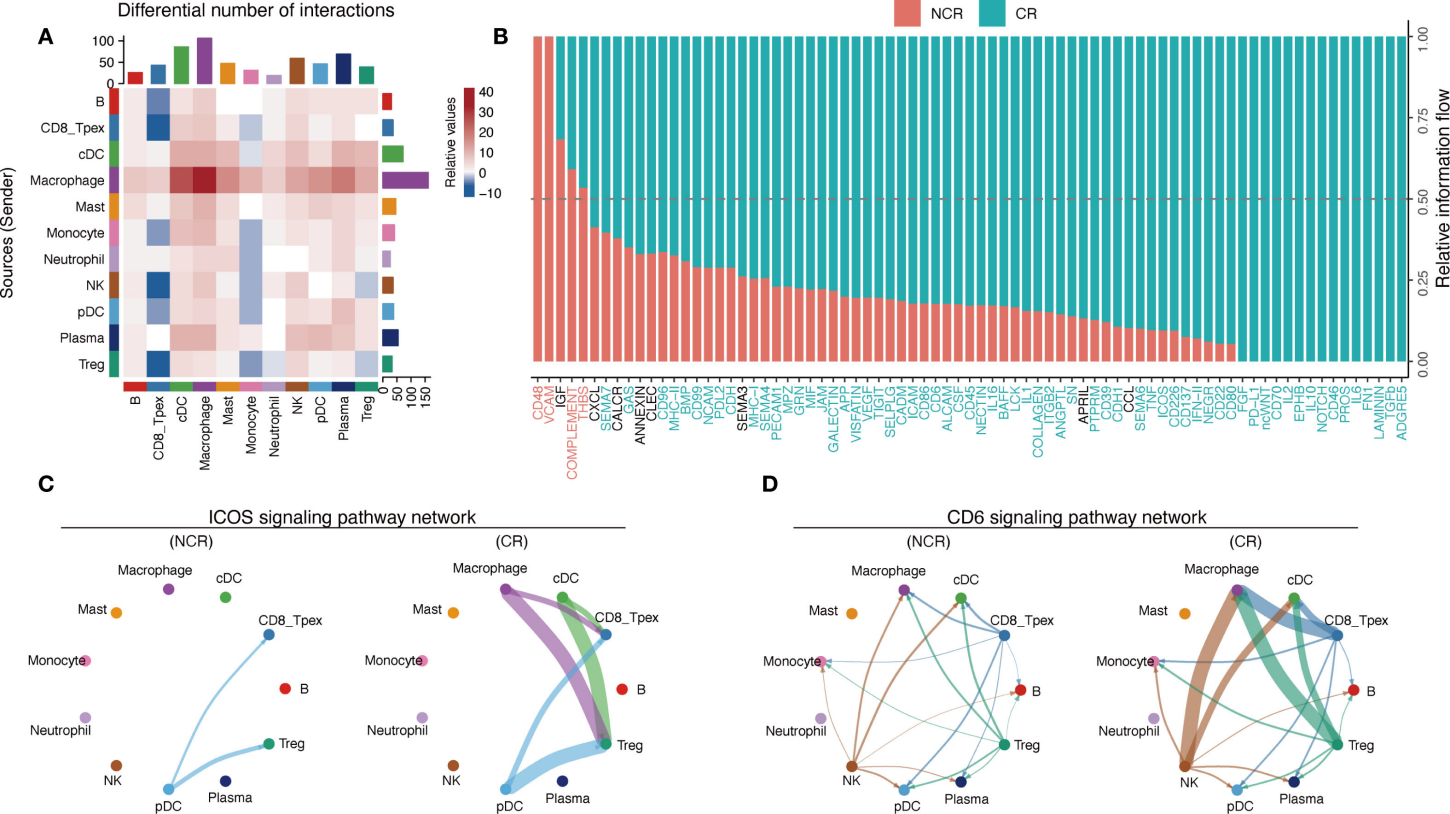
Cell–cell communication landscape between T_pex_ and other immune subsets. **(A)** Total number of ligand–receptor interactions among cell types in NCR and CR groups; communication networks analyzed separately. **(B)** Stacked bar plot representing cumulative information flow contributed by each signaling pathway; dashed line indicates 50% cumulative signaling threshold. **(C, D)** Circle plots depicting representative ligand–receptor signaling pathways (CD6 and ICOS) comparing NCR and CR groups.

Building on the observed differences in the proportion and trajectory of T_pex_, we next assessed whether the microenvironmental signaling milieu differed between CR and NCR groups. Analysis of ligand–receptor interactions revealed that macrophages, plasma cells, and cDCs exhibited substantial differences in incoming signaling, while cDCs and macrophages showed differential outgoing signals (**Figure 5A**). CD8_T_pex_ cells in CR patients displayed enhanced outgoing interactions with macrophages and cDCs, implying their involvement in modulating the immune microenvironment to favor therapeutic response. Conversely, CD8_T_pex_ cells in CR patients received attenuated signals from T_reg_ and NK cells, suggesting a reduced immunosuppressive or cytotoxic regulatory influence.

We further evaluated the overall information flow within the TME. Most signaling pathways enriched in CR were intimately linked to immune activation, including pathways mediating T-cell activation (e.g., CD86, CD137), cytokine signaling (e.g., IL-2, IFN-γ), antigen presentation (e.g., MHC-II), as well as immune cell adhesion and trafficking (e.g., ICAM, SEMA4) (**Figure 5B**). These findings suggested a more immunologically active and coordinated microenvironment in CR patients.

Notably, the CD6 signaling pathway, which modulates T-cell activation, proliferation, and trafficking via interaction with its ligand ALCAM, was significantly upregulated in communications between CD8_T_pex_ cells and macrophages as well as cDCs (45) (**Figure 5C**). This enhanced CD6 signaling likely reflected an elevated activation status within the CR group. The ICOS pathway, known to regulate T helper cell responses and support adaptive immunity, was selectively activated in the interactions between CD8_T_pex_ and macrophages, as well as between CD8_T_pex_ and cDCs in CR patients. (**Figure 5D**).

**Figure 6:**
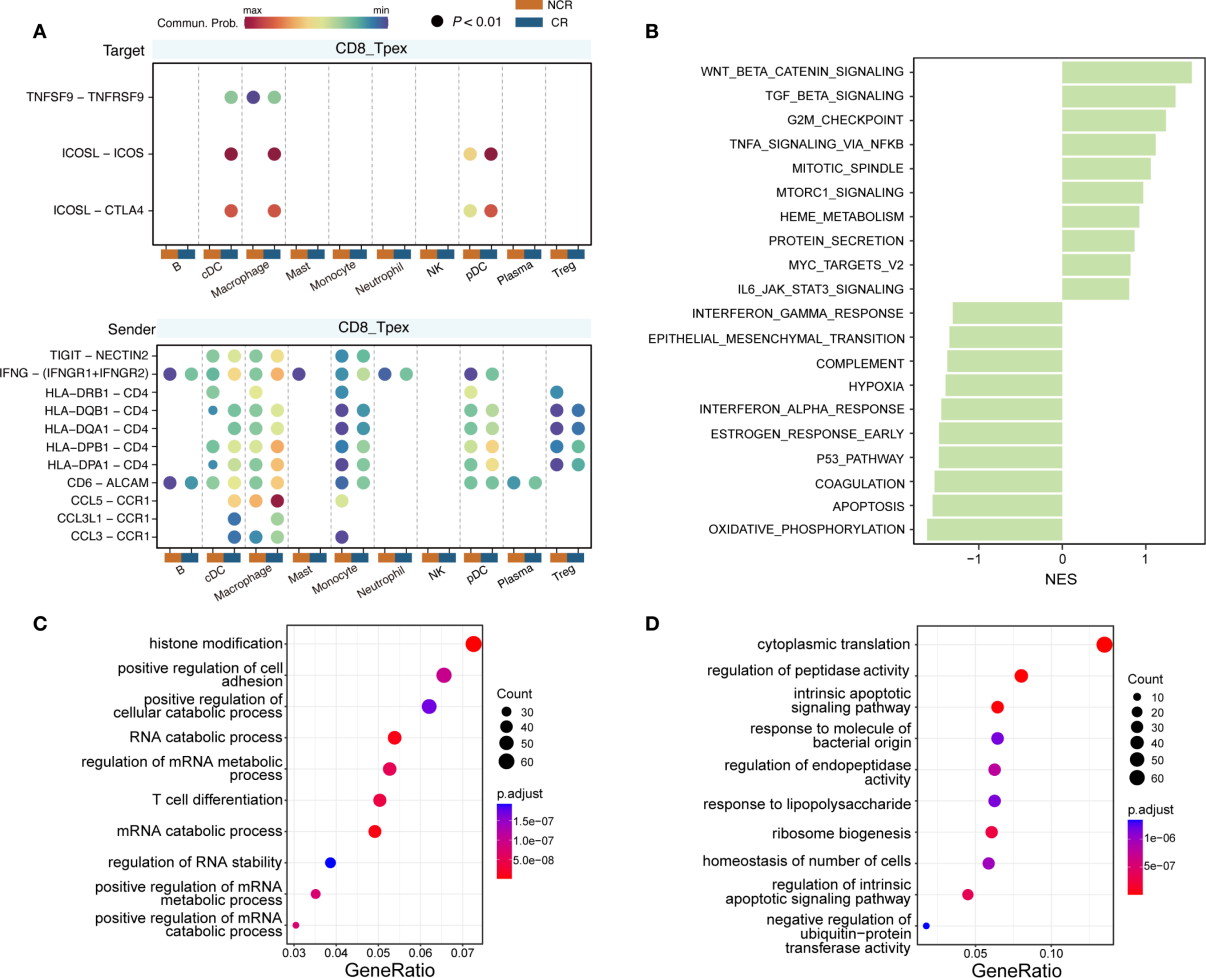
Functional interpretation of T_pex_ signaling by ligand–receptor and pathway enrichment analyses. **(A)** Upregulated ligand–receptor interactions between T_pex_ and other immune subclusters in NCR (coral) vs. CR (blue) groups; dot color reflects communication probability between sender and receiver clusters. **(B)** GSEA of Hallmark pathways was significantly altered (FDR q < 0.05) in CR vs. NCR groups; right: upregulated, left: downregulated pathways shown by normalized enrichment scores (NES). **(C, D)** GO enrichment chord plots linking **(C)** upregulated and **(D)** downregulated genes with their enriched biological processes.

By quantifying ligand–receptor pair ratios, we observed that distinct tissue regions exhibited unique intercellular signaling patterns. Further cell-type-specific analyses revealed that the interaction probability of ICOS-related ligand–receptor pairs, especially TNFSF9–TNFRSF9, in incoming signals to CD8_T_pex_ cells was newly induced or significantly upregulated in CR patients relative to NCR. Additionally, chemokine CCL family members were markedly enriched in the outgoing signaling from CD8_T_pex_ cells in CR patients (**Figure 6A**).

To gain deeper insights into these signaling modifications, pathway enrichment analyses were performed. The findings highlighted key biological processes such as T-cell differentiation, positive regulation of cell adhesion, and histone modification, collectively indicating enhanced immune activation, strengthened intercellular interactions, and possible epigenetic reprogramming of T_pex_ cells in responders (**Figure 6C**). Moreover, numerous RNA metabolic processes were significantly enriched.

Conversely, genes downregulated in CR patients were enriched for processes such as cytoplasmic translation, intrinsic apoptotic signaling, ribosome biogenesis, and responses to bacterial components or lipopolysaccharide (LPS), indicating a possible reduction in protein synthesis burden and stress-related signaling in T_pex_ cells from non-responders (**Figure 6D**). These observations were corroborated by gene set enrichment analysis (GSEA), which further supported elevated transcriptional and post-transcriptional activity in the CR group compared to a more suppressed or dysfunctional transcriptional landscape in the NCR group (**Figure 6B**).

## Discussion

Combining immunotherapy with chemotherapy has demonstrably improved OS and progression-free survival (PFS) in patients with advanced EC. Nevertheless, multiple clinical trials have reported that the CR rate achieved by ICB combined with chemotherapy remains below 20%. In resectable EC, the pathological complete response (pCR) rate following preoperative neoadjuvant ICB therapy is still under 40% (4, 5, 14, 46). ICB efficacy is governed by a complex interplay of determinants, such as tumor mutation burden (TMB), neoantigen load, major histocompatibility complex (MHC) molecule expression, DNA damage repair capacity, and the functional status of T cells (47). A considerable subset of patients fails to benefit from PD-1 blockade, primarily due to the inability to restore the functional competence of exhausted CD8^+^T cells residing in the TME. Therefore, an in-depth characterization of the functional attributes of immune cells infiltrating the EC TME and their responsiveness to immunotherapeutic interventions is essential for driving progress in treatment development (48, 49).

The clinical efficacy of ICB and adoptive cell therapy (ACT) primarily depends on CD8^+^T cells’ ability to effectively eliminate tumor cells. However, chronic antigen exposure—whether during persistent viral infections or tumorigenesis—induces CD8^+^T-cell exhaustion, significantly limiting the success of both immunotherapies (50). T_pex_ cells are defined by their unique co-expression of the molecules Ly108 (surface marker) and TCF7 (transcription factor), both essential for their lineage commitment and maintenance (47). This population exhibits intermediate PD-1 expression levels, serving as a key phenotypic feature that distinguishes them from other exhausted T-cell subsets with divergent functional capacities (51). T_pex_ cells possess self-renewal potential and demonstrate substantial proliferative capacity following PD-1-targeted therapy, thereby sustaining T cell-mediated antitumor immunity (34, 52).

PD-1 blockade has been shown to enhance the proliferative capacity of T_pex_ cells and promote their differentiation into effector-like T_ex_ cells, thereby sustaining effective antitumor immunity (53). Consistent with this mechanistic role, a higher proportion of T_pex_ cells has been reported to correlate with improved clinical outcomes across multiple cancer types. In melanoma, the frequency of T_pex_ cells was positively associated with the duration of response to anti–PD-1 and/or anti–CTLA-4 therapy, and patients with a larger T_pex_ fraction exhibited significantly prolonged PFS (29, 34). Similarly, in non–small cell lung cancer, higher T_pex_ abundance was observed in responders to anti–PD-1 therapy combined with chemotherapy (52). In hepatocellular carcinoma, striking differences between T cell rich tumor lesions from responders and non-responders were identified, with expansion of effective antitumor CD8^+^T cells occurring within tumor microenvironmental niches enriched for T_pex_, CXCL13^+^T_H_ cells, and mregDCs (53). In line with these observations, our previous study in colorectal cancer also demonstrated that T_pex_ infiltration served as a potential predictor of immunotherapy efficacy. Taken together, these findings underscore the clinical relevance of T_pex_ cells as biomarkers of response to PD-1 blockade, and suggest that future therapeutic strategies aimed at enhancing the persistence and expansion of T_pex_ may further improve patient outcomes.

The differentiation and persistence of T_pex_ cells are orchestrated by a network of transcription factors, including TCF7, BCL-6, PRDM1, TOX, IRF4, MYB, alongside cytokines such as type I interferon (IFN-I) and interleukin-27 (IL-27) (54). Significantly, TCF1 upregulates BCL-6 expression, which antagonizes IFN-I signaling to inhibit terminal differentiation of CD8^+^T cells. The absence of TCF1 compromises the sustained responsiveness of T cells without affecting the transcription of exhaustion-related genes (47). Hypoxia and VEGF-A drive the differentiation of terminally exhausted CD8^+^T cells at the expense of the T_pex_ subset, without altering effector cytokine production or GZMB expression (55). Consistent with this mechanism, we found T_pex_ infiltration correlated with tumor size but not with TNM stage. Enlarging tumors are prone to diffusion-limited oxygen supply and abnormal vasculature, resulting in chronic hypoxia, which in turn compromises T_pex_ maintenance. Furthermore, T_pex_ cells reside preferentially in APC-rich niches and tertiary lymphoid structures, the presence of which reflects local tissue architecture rather than the anatomic extent of disease (53, 56). These observations provide a plausible explanation for the differential association of T_pex_ with tumor size and stage. Critically, T_pex_ cells have recently been recognized as distinct predictive biomarkers for ICB responsiveness across various cancer types, highlighting their essential role in the assessment and potentiation of immunotherapeutic outcomes.

DCs serve as pivotal orchestrators of T-cell differentiation by not only presenting antigens through T-cell receptor (TCR) engagement but also by delivering essential cytokines and costimulatory signals that guide T-cell effector fate decisions (57). Reflecting these functions, elevated intratumoral DC abundance has been associated with prolonged OS and enhanced responsiveness to PD-1 blockade, likely due to their critical role in priming CD8^+^T-cell responses in both clinical and preclinical settings (58). Notably, a study in hepatocellular carcinoma has reported that direct interactions between T_pex_ cells and mregDCs facilitate effective T-cell responses (53). In this context, our observation of augmented CD6 signaling and newly activated ICOS signaling pathways between T_pex_ and DCs in CR suggested a more supportive and immunostimulatory microenvironment conducive to T_pex_ activation and effector differentiation. Conversely, the absence of such stimulatory cues in NCR might impede the progression of T_pex_ cells into fully functional effector CD8^+^T cells, a critical process underlying successful PD-1 blockade therapy. In addition to DCs, tumor-associated macrophages (TAMs) serve as crucial antigen-presenting cells (APCs) for presenting tumor antigens to CD8^+^T cells. The role of TAMs in antitumor immunity remains controversial, as they have been associated with both tumor progression and immunosuppression, as well as with potent activation of effector T cells under specific conditions (59, 60). Several studies have implicated TAMs in promoting the differentiation of T_pex_ cells toward a terminally exhausted phenotype, as suggested in a glioblastoma study, where this process may be influenced by their levels of MHC class I molecule expression (61).

The current work confirmed the existence of T_pex_ cells residing in the TME of human ESCC tissues via scRNA-seq analysis, with these cells positioned early in the pseudotime trajectory. Compared to adjacent normal tissues, ESCC samples demonstrated a reduced proportion of T_pex_ cells. Survival analyses revealed that patients exhibiting high T_pex_-cell infiltration experienced significantly improved prognosis relative to those with low infiltration. Subgroup analyses further indicated that among patients with early-stage disease (TNM stages I and II), higher T_pex_ infiltration, elevated T_pex_/CD8^+^T-cell ratios, or increased T_pex_/PD-1^+^CD8^+^T-cell ratios were all associated with modestly better OS. Furthermore, comparison between CR and NCR groups showed that T_pex_ cells were not only more abundant but also transcriptionally more active in CR patients. Collectively, these findings underscored the prognostic value of T_pex_ cells in the ESCC immune microenvironment and highlighted their potential as predictive biomarkers for immunotherapy responsiveness.

In conclusion, our study highlighted T_pex_ cells as powerful prognostic and predictive biomarkers in EC, revealing their distinct infiltration patterns as closely linked to patient outcomes and responsiveness to ICB therapy. These compelling associations not only deepened our understanding of the tumor immune microenvironment but also opened exciting avenues for future research. By leveraging cutting-edge integrative strategies such as single-cell multi-omics and validating findings in larger, well-characterized patient cohorts, we can unlock the full potential of T_pex_ cells. Ultimately, these efforts will pave the way toward more precise, effective immunotherapeutic interventions, offering renewed hope for patients battling this formidable disease.

## Data Availability

The datasets presented in this study can be found in online repositories. The names of the repository/repositories and accession number(s) can be found in the article/**Supplementary Material**.

## Supplementary material

Supplementary Figure 1 Treatment-associated evolution of the tumor immune microenvironment and T-cell phenotypes. **(A)** Subclustering of all cells from dataset HRA003312 identified 16 major cell lineages. **(B)** Heatmap displaying canonical marker gene expression across these cell types. **(C)** Proportion of cells per sample contributing to each cluster, colored by cell type; annotations denote patient, treatment status, and clinical response. **(D)** Proportion of T cells per sample contributing to T-cell subclusters, colored by cell type; annotations as in **(C)**. **(E)** UMAP plot of T cells annotated into nine subclusters.
