# Supplementary figures and images for: Progenitor-exhausted T cell as prognostic indicator in esophageal squamous cell carcinoma: illuminating their key contribution to tumor immunity
Source: Front Immunol. 2025 Sep 26;16:1659077. doi: 10.3389/fimmu.2025.1659077 (PMC12510862; doi:10.3389/fimmu.2025.1659077)

A

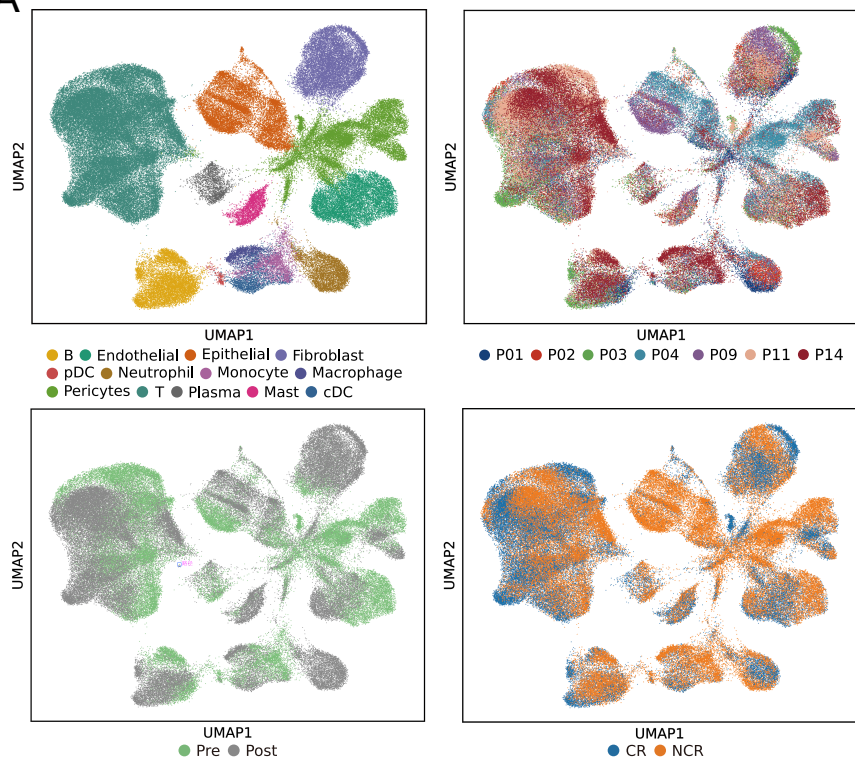

B

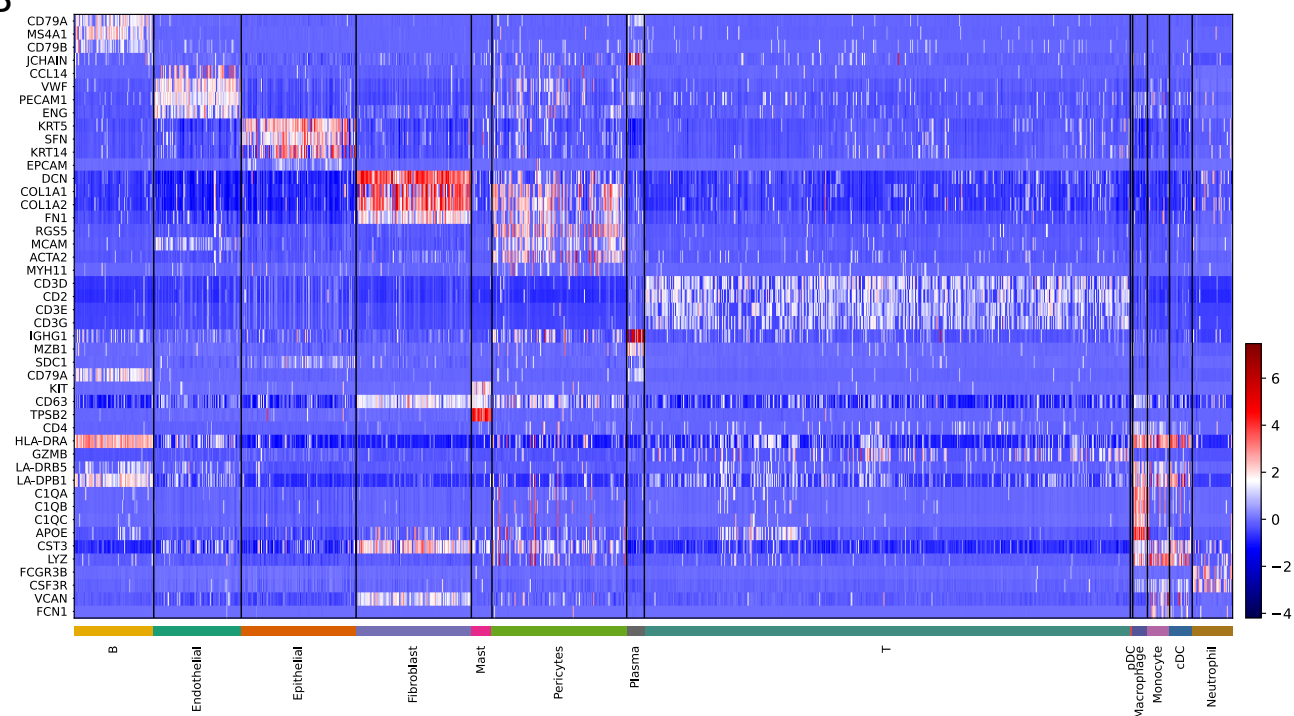

C

All cell types

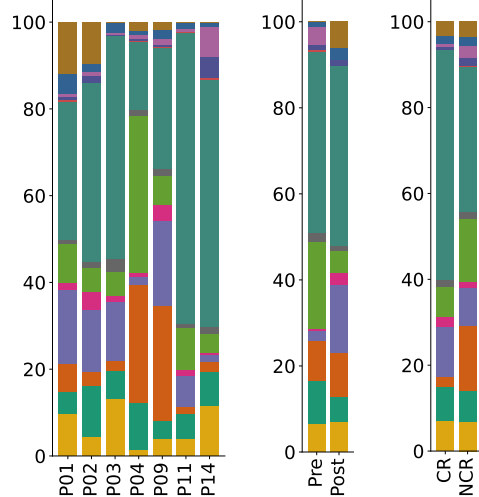

D

Reclustering T cells

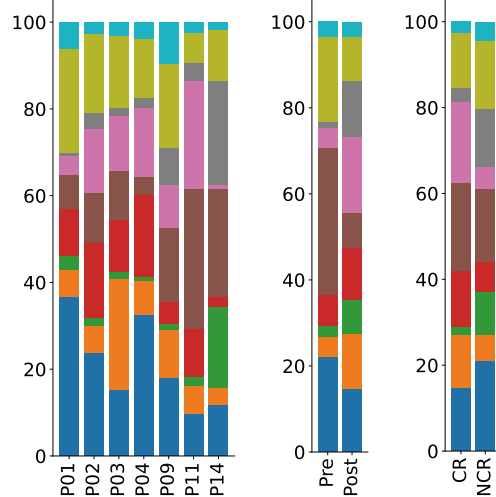

E

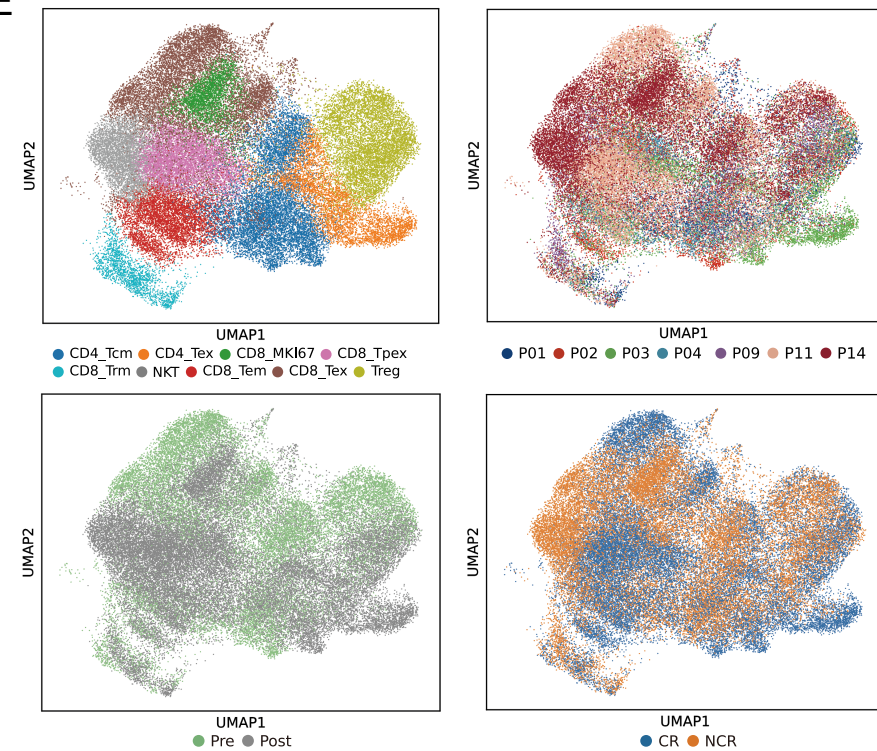

Supplement: Supplementary Figure 1 — Treatment-associated evolution of the tumor immune microenvironment and T-cell phenotypes. (A) Subclustering of all cells from dataset HRA003312 identified 16 major cell lineages. (B) Heatmap displaying canonical marker gene expression across these cell types. (C) Proportion of cells per sample contributing to each cluster, colored by cell type; annotations denote patient, treatment status, and clinical response. (D) Proportion of T cells per sample contributing to T-cell subclusters, colored by cell type; annotations as in (C). (E) UMAP plot of T cells annotated into nine subclusters. [file DataSheet1.pdf]
